# Sequential algorithm analysis to facilitate selective biliary access for difficult biliary cannulation in ERCP: a prospective clinical study

**DOI:** 10.1186/1471-230X-14-30

**Published:** 2014-02-17

**Authors:** Tae Hoon Lee, Soon Oh Hwang, Hyun Jong Choi, Yunho Jung, Sang Woo Cha, Il-Kwun Chung, Jong Ho Moon, Young Deok Cho, Sang-Heum Park, Sun-Joo Kim

**Affiliations:** 1 Division of Gastroenterology, Department of Internal Medicine, Soonchunhyang University School of Medicine, Cheonan Hospital, 23-20 Bongmyung-dong, Cheonan, South Korea; 2 Division of Gastroenterology, Department of Internal Medicine, Soonchunhyang University School of Medicine, Bucheon Hospital, 1174 Jung-dong, Bucheon, South Korea; 3 Division of Gastroenterology, Department of Internal Medicine, Soonchunhyang University School of Medicine, Seoul Hospital, 657 Hannam-dong, Seoul, South Korea

**Keywords:** Difficult biliary cannulation, Precut, Double guidewire cannulation, Pancreatic stent

## Abstract

**Background:**

Numerous clinical trials to improve the success rate of biliary access in difficult biliary cannulation (DBC) during ERCP have been reported. However, standard guidelines or **s**equential protocol analysis according to different methods are limited in place. We planned to investigate a sequential protocol to facilitate selective biliary access for DBC during ERCP.

**Methods:**

This prospective clinical study enrolled 711 patients with naïve papillae at a tertiary referral center. If wire-guided cannulation was deemed to have failed due to the DBC criteria, then according to the cannulation algorithm early precut fistulotomy (EPF; cannulation time > 5 min, papillary contacts > 5 times, or hook-nose-shaped papilla), double-guidewire cannulation (DGC; unintentional pancreatic duct cannulation ≥ 3 times), and precut after placement of a pancreatic stent (PPS; if DGC was difficult or failed) were performed sequentially. The main outcome measurements were the technical success, procedure outcomes, and complications.

**Results:**

Initially, a total of 140 (19.7%) patients with DBC underwent EPF (n = 71) and DGC (n = 69). Then, in DGC group 36 patients switched to PPS due to difficulty criteria. The successful biliary cannulation rate was 97.1% (136/140; 94.4% [67/71] with EPF, 47.8% [33/69] with DGC, and 100% [36/36] with PPS; P < 0.001). The mean successful cannulation time (standard deviation) was 559.4 (412.8) seconds in EPF, 314.8 (65.2) seconds in DGC, and 706.0 (469.4) seconds in PPS (P < 0.05). The DGC group had a relatively low successful cannulation rate (47.8%) but had a shorter cannulation time compared to the other groups due to early switching to the PPS method in difficult or failed DGC. Post-ERCP pancreatitis developed in 14 (10%) patients (9 mild, 1 moderate), which did not differ significantly among the groups (P = 0.870) or compared with the conventional group (P = 0.125).

**Conclusions:**

Based on the sequential protocol analysis, EPF, DGC, and PPS may be safe and feasible for DBC. The use of EPF in selected DBC criteria, DGC in unintentional pancreatic duct cannulations, and PPS in failed or difficult DGC may facilitate successful biliary cannulation.

## Background

Despite the various endoscopic techniques available to facilitate selective biliary access, selective biliary cannulation may be incomplete in 5–10% of patients, even in experienced hands [1,2]. Difficulty in cannulating the biliary ductal system leads to prolonged papillary manipulation, which results not only in pancreatic duct outlet obstruction due to mechanical trauma and edema but also chemical injury due to inadvertent contrast injection into the pancreatic duct. Thus, repeated and prolonged attempts at cannulation may increase the risk of post-ERCP pancreatitis (PEP) [3]. Therefore, various techniques—such as double guidewire-induced cannulation, precut papillotomy, or transpancreatic sphincterotomy with or without placement of a pancreatic stent—have been used to improve cannulation success rates and have shown good clinical results [1,2,4-8].

Recently, Testoni *et al*. [1] suggested an algorithm for biliary cannulation during ERCP based on previous reports. However, a standardized study or protocol analysis has not yet been performed using a sequential method. Step-wise methods may be needed to facilitate selective biliary access without increasing the occurrence of complications such as PEP, and verification of the protocol is also required. Use of a step-wise sequential protocol during difficult biliary cannulation (DBC) may reduce successful cannulation time and inadequate procedure time, so that unexpected complications such as PEP decrease. Additionally, a sequential algorithm may introduce practical guidelines to those who are inexperienced with ERCP.

This prospective study was designed to evaluate a sequential cannulation protocol to facilitate selective biliary access for DBC. The algorithm was based on the sequential performance of an early precut fistulotomy (EPF), double-guidewire cannulation (DGC) technique, and precut after placement of a pancreatic stent (PPS) in failed wire-guided cannulation due to DBC during ERCP.

## Methods

### Study design and patient population

This was a prospective clinical study conducted in a tertiary referral center. A total of 711 consecutive patients with naïve papillae were enrolled from September 2010 to August 2012. Patients who satisfied the following inclusion criteria were enrolled: those with naïve papilla who underwent ERCP for biliary endotherapy, age ≥ 18 years, and agreement to participate in the study. Exclusion criteria were: age < 18 years, successful deep biliary cannulation within 5 min, surgically altered anatomy (Billroth II gastrectomy or Roux-en-Y anastomosis), an indwelling biliary stent, prior biliary or pancreatic sphincterotomy, uncontrolled coagulopathy, and refusal to agree to the study protocol. All patients underwent abdominal ultrasonography, computed tomography, magnetic resonance cholangiopancreatography or endoscopic ultrasonography prior to ERCP. The Institutional Review Board at the Soonchunhyang University Hospital approved this protocol, and all participants provided informed consent.

### Definitions

Difficult biliary cannulation was defined as failure to achieve selective biliary access by wire-guided cannulation despite 5 min of attempted cannulation (cannulation time > 5 min), papillary contacts > 5 times, attempted unintentional pancreatic duct cannulation ≥ 3 times, or hook-nose-shaped papilla (difficult type of cannulation) [6]. Meaningful papillary contact was defined as sustained contact for cannulation between the guidewire-preloaded papillotome and the ampulla of Vater for at least 3–5 seconds of manipulation [6,8,9]. The configuration of a hook-nose-shaped papilla, which is a difficult type to cannulate conventionally, was based on a previous report; papilla shapes were classified into one of four categories: the non-prominent type, wherein the major papilla lacked a marked oral protrusion; the prominent type, with marked oral protrusion of the major papilla; the bulging or hook-nose type, with marked swelling of the oral protrusion; and the distorted type, with an unclassified unusual shape and position [6]. The frequency of papillary contact or unintentional pancreatic duct cannulation was measured by skilled assistant nurses. The procedural time for cannulation was calculated as the interval between the duodenal intubation and initiation of precut or other procedures, whereas the precut procedure time was the interval between initiation of precut and either successful biliary cannulation or termination of the procedure on the video monitoring system. The serum amylase level was measured before ERCP and 24 h after. All complications were classified and graded according to consensus guidelines [10]. PEP was defined as follows; new or worsened abdominal pain with elevation of serum amylase at least three times above the upper normal limits for 24 hours after a procedure that requires at least 2–3 days (mild), 4–10 days (moderate), and more than 10 days (severe) of hospitalization. Hemorrhage was considered clinically significant only if there was clinical (not just endoscopic) evidence of bleeding, such as melena or hematemesis, with an associated decrease of at least 2 g per deciliter in the hemoglobin concentration, or the need for a blood transfusion. Perforation included retroperitoneal or bowel wall perforation documented by any radiographic images.

### Sequential endoscopic procedures

All patients underwent ERCP using a standard duodenoscope (TJF 240 or 260 V; Olympus Optical Co., Ltd., Tokyo, Japan) following an overnight fast. Patients were placed in the prone or lateral position after being sedated with intravenous midazolam (0.05 mg/kg), fentanyl (25–50 μg), and/or propofol (0.5 mg/kg). Prophylactic antibiotics and analgesics were permitted.

### (1) Wire-guided cannulation technique

Selective cannulation of the common bile duct (CBD) was attempted initially using a standard wire-guided cannulation technique. The papillotome was oriented from the 11 to the 12 o’clock positions on the ampulla of Vater and bowed to align it correctly with the bile duct axis. Following minimal insertion of a pull-type papillotome into the papilla, a 0.035-inch guidewire (Hydra Jagwire; Boston Scientific or Tracer Metro; Cook Medical, Winston Salem, NC, USA) was advanced carefully through the CBD under fluoroscopy until it was observed to enter the bile duct. Contrast injection was not attempted routinely before selective placement of the guidewire in the CBD.

### (2) Early precut fistulotomy (EPF)

When wire-guided cannulation failed based on the DBC criteria, including cannulation failure within 5 min of attempted cannulation (cannulation time > 5 min), papillary contact > 5 times, or a hook-nose-shaped papilla, a precut fistulotomy (infundibulotomy) using a needle-knife (Microtome; Boston Scientific, Microvasive, Marlboro, MA, USA) was performed as an rescue method. EPF was initiated over the bulging portion of the papillary roof, extended upwards or downwards, and stopped short of the papillary orifice to avoid the risk of duodenal perforation or injury to the pancreatic sphincter. The bile duct was probed using a guidewire-preloaded needle-knife after making an incision into the intraduodenal segment of the CBD. A precut fistulotomy was performed with a blended electrosurgical current (UES-30 generator; Olympus).

### (3) Double-guidewire cannulation (DGC) technique

If unintentional pancreatic duct cannulation occurred more than 3 times during the initial attempts at wire-guided cannulation, DGC was attempted. We used a papillotome pre-inserted with a 0.035-inch guidewire to facilitate biliary cannulation for DGC. The first guidewire was inserted into the pancreatic duct to at least half of the presumed total length of the pancreatic duct under fluoroscopy. No contrast was injected into the pancreatic duct for the purpose of diagnosis. The papillotome was reinserted along the first guidewire after being reloaded with the second guidewire. The direction of the CBD was towards the 10–11 o’clock position. After successful biliary cannulation was achieved, a biliary sphincterotomy over the guidewire was performed and the guidewire was removed from the pancreatic duct without pancreatic stenting.

### (4) Precut after placement of a pancreatic stent (PPS)

If the sum of papillary contact and unintentional pancreatic duct cannulation exceeded 5 times despite ongoing DGC, PPS was performed. After placement of the pancreatic stent (single 6 or 8 cm-long 5 F pigtail-type, Zimmon; Cook Endoscopy), a cut was made from the orifice of the ampulla of Vater adjacent to the pancreatic stent. Following this, the selective wire-guided cannula method was used for selective biliary access in the same manner. If the pancreatic stent had not migrated spontaneously within 1 week, it was removed endoscopically.

All endoscopic procedures were recorded with a video recording system, and the outcomes were analyzed. All ERCP procedures were performed by a single experienced endoscopist (L.T.H., a workload of 400 ERCPs annually with experience of more than 150 cases of precut). Guidewire manipulation and other endoscopic procedures such as cannulation attempts and time were controlled and measured by skilled assistants with at least 2 years of training (P.S.J., L.J.H., and K.A.R.).

### Summary of the algorithm

**Figure 1:**
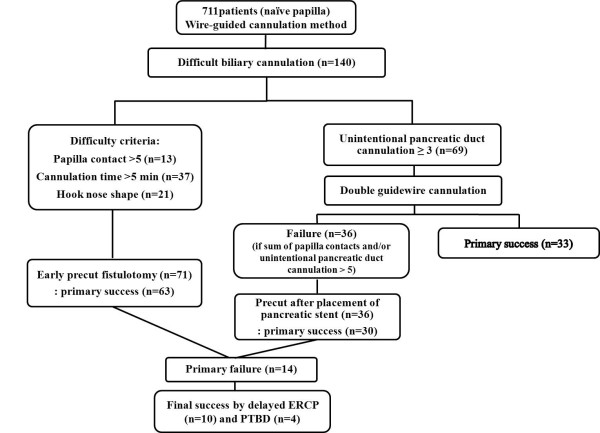
**Algorithm of presented study.** Algorithm for selective biliary access in difficult biliary cannulation during ERCP.

Sequential EPF, DGC, and PPS were performed according to the algorithm (Figure 1). The initial cannulation was attempted using the wire-guided cannulation method. Then, EPF was performed first if primary wire-guided cannulation failed based on the following criteria: papilla contact > 5, cannulation time > 5 min, or a hook-nose-shaped papilla. Next, DGC was attempted if unintentional pancreatic duct cannulation occurred more than 3 times. Third, PPS was attempted if the sum of papilla contact and unintentional pancreatic duct cannulation exceeded five times during the trial of DGC. If selective biliary cannulation failed even after the above procedures, we performed a second ERCP after 2 days or subsequent percutaneous transhepatic biliary drainage (PTBD) depending on the patient’s condition, such as sepsis or severe cholangitis.

### Statistical analysis

The mean, standard deviation (SD), and range were used to summarize the data for continuous variables, and percentages were used for categorical variables. Fisher’s exact test or *χ*^2^-test was used to identify associations between categorical variables. Statistical significance between the groups was tested by one-way analysis of variance. A *P*-value < 0.05 was considered to indicate statistical significance. The statistical analysis was carried out using SPSS ver. 14.0 (SPSS, Chicago, IL, USA).

## Results

A total of 711 patients with naïve papilla underwent therapeutic ERCP. The DBC rate among these patients was 19.6% (140/711). In total, 140 patients with DBC underwent sequential EPF, DGC, and PPS according to the algorithm (Figure 1). The baseline patient demographics and clinical indications for the procedure at the time of inclusion are listed in Table 1. The average age (standard deviation [SD]) was 65.6 (13.9) years, and 70 patients were male. The most common indication for therapeutic ERCP was choledocholithiasis, which occurred in 47.8% (67/140) of the patients. EPF was performed in 71 (50.7%) patients. Initially, DGC was attempted in 69 (59.3%) patients, and then 36 (25.7%) of the patients in whom DGC failed subsequently underwent PPS. No significant difference in baseline characteristics was found, with the exception of age (Table 2).

**Table 1:** Patients characteristics

No.	140
	65.61 (13.93)
	70/70
Age, mean (SD)	
Sex (M/F)	
Indications	
CBD stones	
Acute cholecystitis	67 (47.8)
Malignancy	20 (14.2)
Cholangiocarcinoma	
Pancreas cancer	
Ampullary malignancy	
Metastatic cancer	
CBD dilatation	20 (14.2)
Bile leak	10 (7.1)
Chronic pancreatitis	6 (4.2)
Choledochal cyst	2 (1.4)
Gallstone pancreatitis	3 (2.1)
Parasite	1 (0.7)
Sphincter of Oddi dysfunction	3 (2.1)
	4 (2.8)
	1 (0.7)
	1 (0.7)
	2 (1.4)

*SD* standard deviation, *CBD* common bile duct.

**Table 2:** Baseline outcomes and cannulation time in each groups

	**EPF**	**DGC**	**PPS**	**Total**	***P *** **value**
**No. (%)**	71 (50.7)	33 (23.6)	36 (25.7)	140	
**Age, mean (SD)**	65.6 (14.3)	61.1 (15.4)	69.6 (12.8)		0.048
**Sex (M/F)**	37/34	14/19	19/17		0.608
**PAD (Type I/II/III)**	11 (2/9/0)	4 (0/4/0)	11 (0/10/1)		0.146
**Papillary contact, mean (SD)**	3.6 (2.99)	5.4 (1.39)	4.6 (0.76)		0.001
**Unintentional pancreatic duct cannulation,**	16	33	36		
**Frequency, mean (SD)**	1.19 (0.4)	3.36 (1.05)	5.94 (0.33)	40 (1.94)	<0.01
**Pancreatic contrast injection**	2	1	2		0.757
**Procedure timex, sec (SD)**					
**Initial cannulation time**	262.3 (109.2)		253.6 (35.5)	259.4 (91.1)	0.644
**Precut time**	339.8 (342.4)		452.3 (469.1)	377.7 (391.1)	0.161
**Total cannulation time**	559.4 (412.8)	314.8 (65.2)	706.0 (469.4)	559.7 (403.6)	< 0.05

*SD* standard deviation, *PAD* periampullary diverticulum, *EPF* early precut fistulotomy, *DGC* double-guidewire cannulation, *PPS* precut after placement of a pancreatic stent.

### Procedure outcomes

The mean procedural time (SD) for successful biliary access was 559.7 (403.6) seconds in the enrolled patients with DBC. The selective successful biliary cannulation time was shorter in the DGC group than in the other groups (314.8 ± 65.2 seconds in DGC, 559.4 ± 412.8 seconds in EPF, and 706.0 ± 469.4 seconds in PPS; *P* < 0.05). No significant difference in successful cannulation time between EPF and PPS was found (*P* = 0.127) (Table 2). The success rate of selective biliary cannulation was 90% (126/140) for the first attempt (EPF, 88.7%; DGC, 47.8%; PPS, 83.3%). The first attempted successful DGC rate was relatively low (47.8%; 33/69), but involved a relatively short cannulation time as difficult DGC was early switched to the PPS method, according to the algorithm in difficult DGC. Second attempts performed 2 days later were successful in 10 (83.3%) of 12 patients. The overall successful cannulation rate was 97.1% (136/140; EPF, 94.4%; DGC, 47.8%; PPS, 100%; *P* < 0.001). Technical failure occurred in only 2.8% (4/140), all of which were in the EPF group (Table 3). These four patients had severe strictures of the bile duct due to metastatic malignancy (n = 1), ampulla of Vater cancer (n = 1), or pancreas head cancer (n = 2). A second attempt was successful in four of six in the EPF group. Two patients underwent PTBD due to cholangitis and sepsis after the first attempt, and the other two patients underwent PTBD following failure of the second attempt.

**Table 3:** Technical success and complications

	**EPF**	**DGC**	**PPS**	**Total**	***P *** **value**
**No. (%)**	71 (50.7)	33 (23.6)	36 (25.7)	140	
**Success of biliary cannulation**	67/71 (94.4)	33/69 (47.8)*	36/36 (100)	136/140 (97.1)	< 0.001
**First attempt**	63 (88.7)	33 (47.8)	30 (83.3)	126/140 (90)	
**Second attempt**	4/6 (66.6)	0	6/6 (100)	10/12 (83.3)	
**Failure**	4 (5.6)^†^	0	0	4/140 (2.8)	
**Asymptomatic hyperamylasemia**	5 (7)	3 (9)	2 (5.6)	10 (7.1)	0.849
**PEP (mild/moderate/severe)**	6/1/0 (9.85)	4/0/0 (12)	3/0/0 (8.3)	14 (10)	0.870
**Bleeding**	1 (1.4)	1 (3)	2 (5.6)	4 (2.8)	0.476
**Perforation**	0	0	0	0	

*Among 69 patients who underwent an initial DGC, 36 patients switched to PPS due to difficulty criteria.

^†^Percutaneous transhepatic biliary drainage after second attempt (n =2) and first attempt (n = 2).

*PEP* post-ERCP pancreatitis.

### Procedure-related complications

Post-ERCP pancreatitis developed in 14 patients (10%), and minor bleeding occurred in four (2.8%). PEP was mild except one moderate case of pancreatitis, and no significant differences among the three groups were found (*P* = 0.870). There was no significant difference in the PEP rate between the DBC and remaining conventional group, which was collected during the same study period (10% *vs*. 6.3%, 36/571; *P* = 0.125). In the multivariate analysis, female gender was a risk factor for PEP (odds ratio, 4.16; 95% confidence interval, 1.108–15.645, *P* = 0.035) (Table 4). All complications including bleeding were managed conservatively without mortality.

**Table 4:** Post-ERCP pancreatitis related factors

**Variable**	**Univariate ananlysis *p *** **value**	**Multivariate analysis *p *** **value (adjusted OR, 95% CI)**
Female gender	0.050	0.035 (4.16, 1.108-15.645)
Sphincter of Oddi dysfunction	0.187	-
Papillary contact > 5	0.345	-

*OR* odds ratio, *CI* confidence interval.

## Discussion

Testoni *et al*. [1] suggested an algorithm to facilitate biliary access for DBC during ERCP based on reported studies. However, large-scale follow-up study or protocol analysis of the standardized algorithm may be limited in place. Difficulty in selective biliary cannulation leads to prolonged papillary manipulation and procedure times that may increase procedure-related complications, particularly PEP. In these cases, various biliary cannulation techniques—such as precut, DGC technique, or transpancreatic sphincterotomy with or without placement of a pancreatic stent—have been reported [1,11].

In this study, we planned to evaluate the efficacy of a step-wise sequential cannulation protocol for DBC. Initially, we attempted the wire-guided cannulation method to facilitate biliary access and reduce PEP, based on previous reports [12-14]. Among procedure-related factors, selective cannulation of the CBD by inserting a guidewire might lead to fewer complications than conventional methods that use contrast injection to access the bile duct, although the results of studies on the usefulness of wire-guided cannulation are conflicting [13-15]. However, with the DBC criteria, despite wire-guided cannulation, we attempted the precut fistulotomy technique relatively early, except for cases of unintentional pancreatic duct cannulation. A study of precuts in DBC reported that endoscopist experience with the precut procedure was important, particularly in experienced hands, no increasing the number of complications [6,7,16-18]. We used stricter DBC criteria to prevent frequent papillary contacts causing PEP (5-min cannulation time and 5 attempts at papillary contact) compared with a study which defined DBC as failure to achieve biliary access despite 10 min of attempted cannulation or more than 5 attempted unintentional pancreatic duct cannulations [6,19]. Of the DBC criteria, unintentional pancreatic duct cannulation more than 3 times indicated DGC, and subsequent PPS was attempted without delay when DGC failed, according to the algorithm (Figure 1). We also adapted the configuration criteria for the ampulla of Vater. We attempted an EPF for a hook-nose-shaped papilla, which may be expected to be difficult to cannulate due to the anatomical position of the ampullary orifice. Consequently, we applied the step-wise techniques early following the stricter DBC criteria, resulting in a higher technical success rate and reduced successful cannulation time compared with a previous our report (9.2 ± 7.3 minutes in our study *vs*. 16.9 ± 9.1 minutes, *P* < 0.05) [6].

With our algorithm, first we used wire-guided cannulation without contrast injection as the initial cannulation method. Using wire-guided cannulation before contrast injection might be controversial, although meta-analyses have shown that a higher biliary cannulation success rate and lower risk of PEP resulted from avoiding contrast injection into the pancreas [20,21]. Nevertheless, more techniques might be needed for difficult cannulation cases, such as a precut papillotomy with or without a pancreatic stent or the DGC technique.

Second, a needle-knife precut papillotomy can be used to increase selective biliary access during DBC. Both the needle-knife precut procedure and ongoing repeated attempts at cannulation of the ampulla of Vater, regardless of whether they succeed, have been reported to be independent procedure-related risk factors for PEP, together with pancreatic duct cannulation and contrast injection. Therefore, the needle-knife precut papillotomy should be reserved for cases in which all other methods have failed and should be performed by experienced endoscopists [10]. However, Huibregtse *et al*. [16] demonstrated that early implementation of a precut increased successful biliary access on the first attempt, as well as the overall success, and reduced the complication rate to 11.8% (pancreatitis, 0.5%). A recent meta-analysis also showed that the precut technique reduces the risk of PEP significantly [22]. Thus, the early use of a precut fistulotomy in DBC, as in the presented algorithm, might facilitate cannulation without increasing PEP, especially in experienced hands.

Third, regarding the DGC technique, we attempted DGC early for selective biliary access if unintentional pancreatic duct cannulation was repeated. Once a guidewire was placed in the pancreatic duct, it was used to either insert a small stent into the pancreatic duct to facilitate biliary cannulation, or a pancreatic stent was left after the procedure, to prevent pancreatitis in cases with patient- or technique-related risks for this complication. Accordingly, when unintentional pancreatic duct cannulation was repeated 3 or more times, a guidewire was left in the main pancreatic duct and cannulation was continued using the DGC technique. Placement of a guidewire deep in the main pancreatic duct might help to open the papillary orifice and straighten the common pancreaticobiliary channel, facilitating biliary access, and mechanically closing the pancreatic orifice, facilitating cannulation of the bile duct using a second device alongside the pancreatic wire [1,11]. Several studies have reported the efficacy and safety of the DGC technique in DBC [8,23,24]. The technical success rate is 47–93% in patients who fail standard biliary cannulation [4,5,25]. However, the rates of procedural-related complications (*i.e*., PEP) range from no difference to a higher incidence compared with a conventional cannulation group [5,23].

Fourth, to prevent mechanical trauma due to frequent guidewire insertion or papillary contacts, with frequent papillary contacts or repeated unintentional pancreatic duct cannulation despite DGC, a precut from the ampullary orifice was performed following insertion of a prophylactic pancreatic stent. Therefore, in our results the actual first successful DGC cannulation rate was relatively low (47.8%; 33/69) due to early sequential placement of a pancreatic stent in difficult DGC following the protocol. Early pancreatic stenting is useful to reduce the risk of pancreatitis in high-risk conditions, as well as to facilitate biliary cannulation [26]. Once the stent is placed, attempts can be made to cannulate the CBD above the stent, using a sphincterotome and the guidewire-assisted technique. However, a stent might make biliary cannulation with a sphincterotome troublesome; a precut can be easier in these cases. Two studies suggested that a needle-knife biliary sphincterotomy over a pancreatic stent is safer than a conventional pull-type biliary sphincterotomy without a stent in patients with sphincter of Oddi dysfunction [27,28]. Multiple manipulations of the ampulla of Vater before pancreatic stenting or frequent contrast injections might be a risk factor. Accordingly, reducing papillary manipulation or guidewire insertion might be important to prevent PEP. Based on this background, we started the precut at the papillary orifice after pancreatic stent placement to avoid unnecessary manipulations.

Finally, endoscopist experience may be a key factor for preventing pancreatitis during ERCP in average-risk subjects, based on the technique used for cannulation of the ampulla of Vater, the type of device and technique used to achieve deep biliary cannulation, the timing of precut, and the decision on whether and how to insert a pancreatic stent at the end of the procedure. Our study protocol was performed by one experienced endoscopist; trainees were not involved.

Based on these algorithm characteristics, we expected that sequential step-wise cannulation by an expert may reduce procedural-related complications and cannulation time. Although our study was not comparative, our results demonstrate a reduction in successful cannulation time compared with our previous report [6]. However, no differences in the technical success rate of cannulation or procedural-related complications were found. The reported successful cannulation rate is 93.7% and rates of complications including pancreatitis, bleeding, perforation, and cholangitis were 5–19% for precut fistulotomy [6,18,29,30]. In the present study, the overall complication (12.8%) and PEP (10%) rates were similar to those reported previously, and the overall success rate was similar at 97.1%. Female gender was a risk factor for PEP. These complications were treated conservatively without mortality.

The limitations of this study include its non-direct comparative design and single-center nature. Second, this protocol analysis was also performed by a single operator. It may be difficult to suggest a fixed algorithm for every endoscopist due to differences in levels of experience and skill. In addition, each patient and papilla is also different. Although the endoscopic procedures were performed by an expert and we invoked the step-wised protocol early, no difference in PEP or success rates among the groups was found, which might have been due to the relatively small number of participants or the low-risk cohort. Third, while the vast majority of cannulations will be successful with either standard or DGC or even standard dye-based techniques, relatively early step-wise techniques included a needle-knife which might be difficult to agree with. However, the presented algorithm showed that the early adoption of step-wise techniques may be effective and safe in difficult situations, which may overcome the hesitance to perform an advanced technique when persistence with safer techniques is likely to succeed. Further prospective large-scaled comparative studies in different geographical areas and including high-risk cohorts are needed.

## Conclusions

This protocol analysis study was the first trial to evaluate a sequential algorithm for DBC during ERCP. Based on the protocol analysis EPF, DGC, and PPS may be safe and feasible methods in DBC for reducing cannulation time without increasing complications. The use of EPF in selected DBC criteria, DGC in repeated unintentional pancreatic duct cannulations, and PPS in failed or difficult DGC may facilitate successful biliary cannulation. However, further large-scale, multicenter studies are needed to establish the validity of both the algorithm and the guidelines.

## Abbreviations

DBC: Difficult biliary cannulation; EPF: Early precut fistulotomy; DGC: Double-guidewire cannulation; PPS: Precut after placement of a pancreatic stent; CBD: Common bile duct; PEP: Post-ERCP pancreatitis.

## Competing interest

The authors declare that they have no conflict of interest.

## Authors’ contributions

**Guarantor of the article:** THL, MD, PhD.

Conception and design of the study: THL; Generation, assembly, analysis of data, interpretation of data and statistical analysis: SOH, THL, YHJ, HJC, SWC, IKC; The critical revision of the manuscript for important intellectual content: JHM, YDC, SHP, SJK; and drafting of the paper: SOH, THL. Approval of the final draft submitted was taken by all authors. All coauthors read and approved the manuscript and provided substantial suggestions.

## Pre-publication history

The pre-publication history for this paper can be accessed here:

http://www.biomedcentral.com/1471-230X/14/30/prepub

## References

[B1] Testoni PA Testoni S Giussani A Difficult biliary cannulation during ERCP: how to facilitate biliary access and minimize the risk of post-ERCP pancreatitis Dig Liver Dis 2011 43 596 603 10.1016/j.dld.2011.01.019 21377432

[B2] Cortas GA Mehta SN Abraham NS Barkun AN Selective cannulation of the common bile duct: a prospective randomized trial comparing standard catheters with sphincterotomes Gastrointest Endosc 1999 50 775 779 10.1016/S0016-5107(99)70157-4 10570335

[B3] Freeman ML DiSario JA Nelson DB Fennerty MB Lee JG Bjorkman DJ Overby CS Aas J Ryan ME Bochna GS Shaw MJ Snady HW Erickson RV Moore JP Roel JP Risk factors for post-ERCP pancreatitis: a prospective, multicenter study Gastrointest Endosc 2001 54 425 434 10.1067/mge.2001.117550 11577302

[B4] Angsuwatcharakon P Rerknimitr R Ridtitid W Ponauthai Y Kullavanijaya P Success rate and cannulation time between precut sphincterotomy and double-guidewire technique in truly difficult biliary cannulation J Gastroenterol Hepatol 2012 27 356 361 10.1111/j.1440-1746.2011.06927.x 21916994

[B5] Herreros De Tejada A Calleja JL Díaz G Pertejo V Espinel J Cacho G Jiménez J Millán I García F Abreu L UDOGUIA-04 Group: **Double**-**guidewire technique for difficult bile duct cannulation**: **a multicenter randomized**, **controlled trial** Gastrointest Endosc 2009 70 700 709 10.1016/j.gie.2009.03.031 19560764

[B6] Lee TH Bang BW Park SH Jeong S Lee DH Kim SJ Precut fistulotomy for difficult biliary cannulation: is it a risky preference in relation to the experience of an endoscopist? Dig Dis Sci 2011 56 1896 1903 10.1007/s10620-010-1483-z 21082346

[B7] Fukatsu H Kawamoto H Harada R Tsutsumi K Fujii M Kato H Hirao K Nakanishi T Mizuno O Ogawa T Ishida E Okada H Sakaguchi K Quantitative assessment of technical proficiency in performing needle-knife precut papillotomy Surg Endosc 2009 23 2066 2072 10.1007/s00464-008-9969-x 18528622

[B8] Maeda S Hayashi H Hosokawa O Dohden K Hattori M Morita M Kidani E Ibe N Tatsumi S Prospective randomized pilot trial of selective biliary cannulation using pancreatic guide-wire placement Endoscopy 2003 35 721 724 12929017 10.1055/s-2003-41576

[B9] Bailey AA Bourke MJ Kaffes AJ Byth K Lee EY Williams SJ Needle-knife sphincterotomy: factors predicting its use and the relationship with post-ERCP pancreatitis (with video) Gastrointest Endosc 2010 71 266 271 10.1016/j.gie.2009.09.024 20003969

[B10] Cotton PB Eisen GM Aabakken L Baron TH Hutter MM Jacobson BC Mergener K Nemcek A Jr Petersen BT Petrini JL Pike IM Rabeneck L Romagnuolo J Vargo JJ A lexicon for endoscopic adverse events: report of an ASGE workshop Gastrointest Endosc 2010 71 446 454 10.1016/j.gie.2009.10.027 20189503

[B11] Freeman ML Guda NM ERCP cannulation: a review of reported techniques Gastrointest Endosc 2005 61 112 125 10.1016/S0016-5107(04)02463-0 15672074

[B12] Lee TH Park Do H Park JY Kim EO Lee YS Park JH Lee SH Chung IK Kim HS Park SH Kim SJ Can wire-guided cannulation prevent post-ERCP pancreatitis? A prospective randomized trial Gastrointest Endosc 2009 69 444 449 10.1016/j.gie.2008.04.064 19007927

[B13] Artifon EL Sakai P Cunha JE Halwan B Ishioka S Kumar A Guidewire cannulation reduces risk of post-ERCP pancreatitis and facilitates bile duct cannulation Am J Gastroenterol 2007 102 2147 2153 10.1111/j.1572-0241.2007.01378.x 17581267

[B14] Lella F Bagnolo F Colombo E Bonassi U A simple way of avoiding post-ERCP pancreatitis Gastrointest Endosc 2004 59 830 834 10.1016/S0016-5107(04)00363-3 15173796

[B15] Vandervoort J Soetikno RM Tham TC Wong RC Ferrari AP Jr Montes H Roston AD Slivka A Lichtenstein DR Ruymann FW Van Dam J Hughes M Carr-Locke DL Risk factors for complications after performance of ERCP Gastrointest Endosc 2002 56 652 656 10.1016/S0016-5107(02)70112-0 12397271

[B16] Huibregtse K Katon RM Tytgat GN Precut papillotomy via fine-needle knife papillotome: a safe and effective technique Gastrointest Endosc 1986 32 403 405 10.1016/S0016-5107(86)71921-4 3803839

[B17] Kasmin FE Cohen D Batra S Cohen SA Siegel JH Needle-knife sphincterotomy in a tertiary referral center: efficacy and complications Gastrointest Endosc 1996 44 48 53 10.1016/S0016-5107(96)70228-6 8836716

[B18] O’Connor HJ Bhutta AS Redmond PL Carruthers DA Suprapapillary fistulosphincterotomy at ERCP: a prospective study Endoscopy 1997 29 266 270 10.1055/s-2007-1004187 9255529

[B19] Kaffes AJ Sriram PV Rao GV Santosh D Reddy DN Early institution of pre-cutting for difficult biliary cannulation: a prospective study comparing conventional vs. a modified technique Gastrointest Endosc 2005 62 669 674 10.1016/j.gie.2005.05.022 16246677

[B20] Cennamo V Fuccio L Zagari RM Eusebi LH Ceroni L Laterza L Fabbri C Bazzoli F Can a wire-guided cannulation technique increase bile duct cannulation rate and prevent post-ERCP pancreatitis?: A meta-analysis of randomized controlled trials Am J Gastroenterol 2009 104 2343 2350 10.1038/ajg.2009.269 19532133

[B21] Cheung J Tsoi KK Quan WL Lau JY Sung JJ Guidewire versus conventional contrast cannulation of the common bile duct for the prevention of post-ERCP pancreatitis: a systematic review and meta-analysis Gastrointest Endosc 2009 70 1211 1219 10.1016/j.gie.2009.08.007 19962504

[B22] Gong B Hao L Bie L Sun B Wang M Does precut technique improve selective bile duct cannulation or increase post-ERCP pancreatitis rate? A meta-analysis of randomized controlled trials Surg Endosc 2010 24 2670 2680 10.1007/s00464-010-1033-y 20414680

[B23] Gyokeres T Duhl J Varsanyi M Schwab R Burai M Pap A Double guide wire placement for endoscopic pancreaticobiliary procedures Endoscopy 2003 35 95 96 10.1055/s-2003-36403 12510238

[B24] Ito K Fujita N Noda Y Kobayashi G Obana T Horaguchi J Takasawa O Koshita S Kanno Y Pancreatic guidewire placement for achieving selective biliary cannulation during endoscopic retrograde cholangio-pancreatography World J Gastroenterol 2008 14 5595 5600 10.3748/wjg.14.5595 18810780 PMC2746349

[B25] Draganov P Devonshire DA Cunningham JT A new technique to assist in difficult bile duct cannulation at the time of endoscopic retrograde cholangiopancreatography JSLS 2005 9 218 221 15984715 PMC3015580

[B26] Ito K Fujita N Noda Y Kobayashi G Obana T Horaguchi J Takasawa O Koshita S Kanno Y Ogawa T Can pancreatic duct stenting prevent post-ERCP pancreatitis in patients who undergo pancreatic duct guidewire placement for achieving selective biliary cannulation? A prospective randomized controlled trial J Gastroenterol 2010 45 1183 1191 10.1007/s00535-010-0268-7 20607310

[B27] Fogel EL Eversman D Jamidar P Sherman S Lehman GA Sphincter of Oddi dysfunction: pancreaticobiliary sphincterotomy with pancreatic stent placement has a lower rate of pancreatitis than biliary sphincterotomy alone Endoscopy 2002 34 280 285 10.1055/s-2002-23629 11932782

[B28] Madacsy L Kurucsai G Fejes R Szekely A Szekely I Prophylactic pancreas stenting followed by needle-knife fistulotomy in patients with sphincter of Oddi dysfunction and difficult cannulation: new method to prevent post-ERCP pancreatitis Dig Endosc 2009 21 8 13 10.1111/j.1443-1661.2008.00819.x 19691794

[B29] Harewood GC Baron TH An assessment of the learning curve for precut biliary sphincterotomy Am J Gastroenterol 2002 97 1708 1712 10.1111/j.1572-0241.2002.05829.x 12135022

[B30] Mavrogiannis C Liatsos C Romanos A Petoumenos C Nakos A Karvountzis G Needle-knife fistulotomy versus needle-knife precut papillotomy for the treatment of common bile duct stones Gastrointest Endosc 1999 50 334 339 10.1053/ge.1999.v50.98593 10462652

